# An Unusual Intestinal Finding in a Patient with ESKD and Abdominal Pain

**DOI:** 10.34067/KID.0000000000000070

**Published:** 2023-03-30

**Authors:** Catarina Mateus, Pedro Luís, Rita Birne

**Affiliations:** Centro Hospitalar Lisboa Ocidental, Nephrology, Lisbon, Portugal

**Keywords:** acid/base and electrolyte disorders, calcium polystyrene sulfonate, hyperkalemia, intestinal perforation, kalimate, kalimate crystals, potassium, potassium binder

## Abstract

**Figure:**
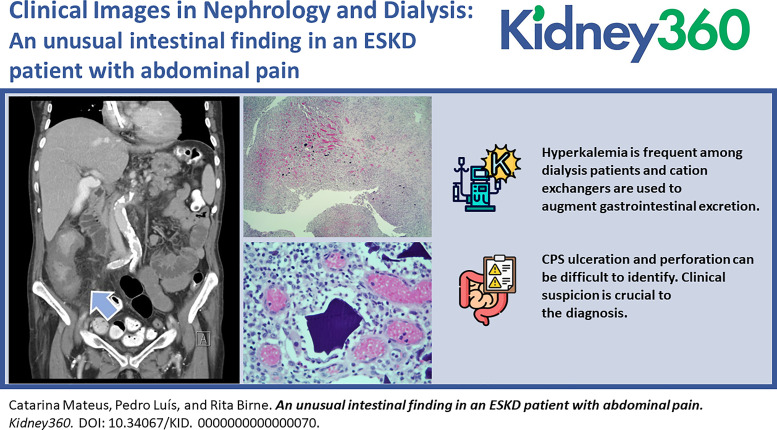

## Case Description

A 57 -year-old Caucasian woman, with ESKD due to HIV-associated nephropathy, on hemodialysis (HD) was admitted for evaluation of abdominal pain, diarrhea, and fever. She was taking clonidine, minoxidil, amiodarone, darunavir/ritonavir/dolutegavir/etravirine, and calcium polystyrene sulfonate (CPS). On physical examination, she had severe abdominal pain with guarding.

**Figure 1:**
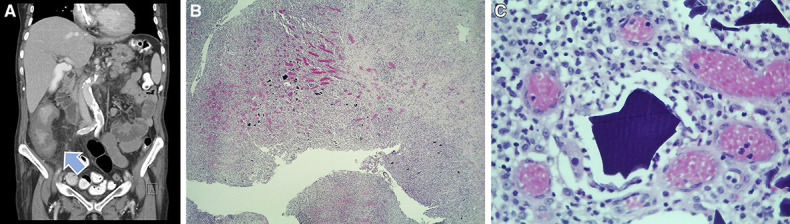
**Imaging and Pathology Study Results.** (A) Abdominal computed tomography revealing concentric circumferential parietal thickening and swelling of the submucosa of the ascending colon and cecum (arrow) with diffuse increase in density of the surrounding fat. (B) Hematoxilin-eosin (40×) histologic section of the bowel wall showing ulceration, granulation tissue, and fibrinogranulocytic peritonitis with readily identifiable intensely basophilic polygonal structures. (C) Hematoxilin-eosin (650×) high power detail of the basophilic structures that reveal a fish scale pattern: kalimate crystals (calcium polystyrene sulfonate crystals).

Laboratory assessment revealed elevated inflammatory markers (C-reactive protein 27.8 mg/dl, white cell count 19×10^9^ with 91.9% neutrophils) and elevated lactate dehydrogenase (302 U/L). Abdominal computed tomography revealed circumferential parietal thickening and swelling of the submucosa of the ascending colon and the proximal half of the transverse colon, with diffuse increase in density of the surrounding fat and small locoregional ganglia (Figure 1A). Blood cultures and fecal cultures, *Clostridium* toxins, and cytomegalovirus viral load were negative. Colonoscopy showed semicircular necrotic ulcers in the right colon. The biopsy of the ulcer fundus revealed extensive erosion and ulceration, with dense fibrinogranulocytic exudate.

The patient was treated with ciprofloxacin, metronidazole, and later piperacillin/tazobactam for suspected infectious colitis, without improvement. The patient developed intestinal subocclusion with persistent pain, elevated inflammatory markers, and intestine distension on abdominal x-ray. She was submitted to exploratory laparotomy, with resection of 30 cm of the small intestine. Macroscopic examination revealed adhesions, with serosa covered by fibrinopurulent exudate, edematous bowel wall, and mucosa with loss of haustration with areas of ulceration. Histologically, there were areas of erosion and ulceration with perforation and fibrinogranulocytic peritonitis, in relation to basophilic crystals, with a fish scale pattern: morphology compatible with kalimate crystals (CPS crystals) (Figure 1, B and C).

After surgery, therapy with CPS was discontinued, and the dietary potassium restrictions were reinforced. The condition resolved without recurrence of symptoms.

## Discussion

Hyperkalemia is a frequent electrolyte imbalance and is associated with increased mortality in patients with chronic kidney disease, including patients with ESKD on HD.^1,2^ Reduction of potassium in the diet, review of dialysis dose and dialysate potassium concentration, and exclusion of access dysfunction are initial measures to approach chronic hyperkalemia in HD patients.^1^ Potassium binders promote gastrointestinal potassium excretion by a cationic exchange to prevent and treat hyperkaliemia.^2^ Despite safety concerns and a lack of randomized evidence for efficacy, sodium polystyrene sulfonate and CPS continue to be the first line of therapy in several countries.^2,3^

Similar to sodium polystyrene sulfonate, CPS has been linked to severe adverse gastrointestinal lesions, such as mucosal edema, ulceration, pseudomembrane formation, and transmural necrosis.^4,5^ In this case, kalimate crystals leading to bowel perforation illustrates how the diagnosis can be delayed because of difficulties in identifying the etiology of colonic lesions. A high index of suspicion is required because withholding the medication as soon as possible is crucial to avoid permanent disability and death. Gastrointestinal adverse effects can happen at any age, and when CPS is prescribed, patients should be closely monitored.^5^

## Teaching Points

- Hyperkalemia is frequent among dialysis patients, and cation exchangers are used to augment gastrointestinal excretion.
- CPS ulceration and perforation can be difficult to identify. Clinical suspicion is crucial to the diagnosis.
